# Eye Explorer: A robotic endoscope holder for eye surgery

**DOI:** 10.1002/rcs.2177

**Published:** 2020-10-10

**Authors:** Dongbo Zhou, Shintaro Kimura, Hayato Takeyama, Daisuke Haraguchi, Yoshihiro Kaizu, Shintaro Nakao, Koh‐Hei Sonoda, Kotaro Tadano

**Affiliations:** ^1^ Institute of Innovation Research Tokyo Institute of Technology Yokohama‐shi Japan; ^2^ School of Engineering Tokyo Institute of Technology Yokohama‐shi Japan; ^3^ Department of Ophthalmology Kyushu University Hospital Fukuoka Japan

**Keywords:** dual‐hand operation, endoscope holder, eye surgery, surgery assistant robot

## Abstract

**Background:**

Holding endoscopes by hand when performing eye surgery reduces the dexterity of the surgeon.

**Methods:**

A robotic endoscope holder called “Eye Explorer” is proposed to hold the endoscope and free the surgeon's hand.

**Results:**

This device satisfies the engineering and clinical requirements of eye surgery. The force for manual operation is less than 0.5 N. The observable ranges inside the patient's eye considering horizontal and vertical perspectives are 118° and 97°, and the motion of the holder does not interfere with the surgeon's hand and other surgical devices. The self‐weight compensation can prevent the endoscope from falling when extra supporting force is released. When comparing the external force exerted on the eye by the Eye Explorer with that in case of manual operation, a decrease of more than 15% can be observed. Moreover, the consumption time of endoscope view adjustment using the Eye Explorer and manual operation does not significantly differ.

**Conclusion:**

The Eye Explorer allows dual‐hand operation, facilitating a successful endoscopic eye surgery.

## 1 INTRODUCTION

Eye surgery has been successfully conducted in clinical trials for several years. Because the small volume and delicate internal structure of the eye present unique challenges to surgeons, surgical assistant robots can play an important role.

Since the initial investigation of robot assistance in eye surgery in the 1980s,^1^ many robotic devices have been developed. These devices can be categorized into two telemanipulation and co‐manipulation systems. When using a telemanipulation system, a slave device reproduces the motion (often scaled‐down) generated by the operator with a master device with a higher operation accuracy. For example, Nasseri et al.^2^ developed a robot system for assistance in ophthalmic surgery, in which the hand tremor of the surgeon can be considerably reduced, resulting in a motion accuracy of the end effector up to 5 μm. Jason et al.^3^ developed a master–slave intraocular robotic interventional surgical system (IRISS), which was the first robotic system to successfully create a round and curvilinear capsulorhexis that is essential for cataract surgery and was considered to be potentially applicable for eye surgery procedures, such as lens removal.^4^ Sakai et al.^5^ developed a master–slave miniature robot for eye surgery. Meanwhile, the Preceyes surgical system developed by Preceyes BV has been demonstrated to safely assist doctors in performing retinal vitreous surgery on humans,^6^ receiving CE marking approval in 2019.

When using a co‐manipulation system, the surgeon directly holds the surgery device connecting to the actuation part to do the operation. The motion of the device is mainly determined by the surgeon, but the actuation part also outputs auxiliary forces to reduce the hand tremors or constrain the motion within a safe area. The Steady‐Hand Robotic System^7^ developed by John Hopkins University, is a representative co‐manipulation system. Patel et al. applied an adaptive control method on the Steady‐Hand Robotic System to make the sclera force or insertion depth of the tooltip be within the pre‐defined safe trajectories.^8^ The KU Leuven robotic system can be operated in both telemanipulation and co‐manipulation modes. By using the KU Leuven robotic system, the first robotic‐assisted endovascular surgery was performed on a human patient in 2018.^9^

Regardless of the operation method, a clear view of the operation field is crucial in case of eye surgery procedures. Microscopes are widely used to obtain a clear view. Some improvements on the microscope were developed for integrating them with a robot‐assisted eye surgery. Inoue et al.^10^ developed a wide‐angle viewing system to provide a wide‐ranging view of the eye. Zhou et al.^11^ developed an image guiding system for a surgeon looking through a microscope. Mukherjee et al.^12^ developed a fast and accurate algorithm that can map the retinal vasculature and localize the retina with respect to the microscope. However, it is difficult to observe the intraocular area that is at the back of the endoscope because of the fixed perspective. Additionally, the condition of the patient's eye may affect the quality of the view. A surgeon may not be able to obtain a clear view if the cornea is muddy or the pupil is shrunken.

Optical coherence tomography (OCT) is used to compensate for the disadvantages associated with the microscope. OCT can be used to obtain detailed images of the operation area inside the eye, and the quality of the image is not affected by the condition of the patient's eye. Thus, some groups have investigated OCT‐integrated robot‐assisted eye surgery. Yu et al. evaluated the efficacy of robot‐assisted microsurgery with OCT guidance.^13^ Chen et al. evaluated an OCT‐guided robot‐assisted cataract removal surgery.^14^ Balicki and Yang respectively evaluated the utility of the OCT‐guided eye surgery with respect to the co‐manipulation robot systems.^15^, ^16^

However, the view range of an OCT image is limited, and the operation of real‐time OCT for view alternation has been reported as ‘complicated’ by surgeons. Furthermore, the performance of the OCT images, considering real‐time situations, is poor because the highest imaging speed is 10–20 volumes/s,^17^ even when using a high‐grade graphics processing unit.

In such cases, using an intraocular endoscope is an alternative choice. The intraocular endoscope can provide real‐time images independent of the condition of the patient's eye. Moreover, the view range, location and zooming capacity of an endoscope can be altered as the surgeon deems necessary. In many cases, endoscopes and microscopes are used together.

Therefore, the usage of an intraocular endoscope in eye surgery has increased, especially in case of the vitrectomy surgery, retinal vein occlusion, retinal detachment and intraocular tumour treatment. However, the adoption of endoscopic eye surgery is still limited despite its benefits. This can be attributed to the fact that the surgeons have to use one hand to hold the endoscope, making it difficult to perform dual‐hand operations. Whereas the operation dexterity of dual‐hand operation is expected in complicated surgeries such as the inner limiting membrane detachment.^18^

**FIGURE 1:**
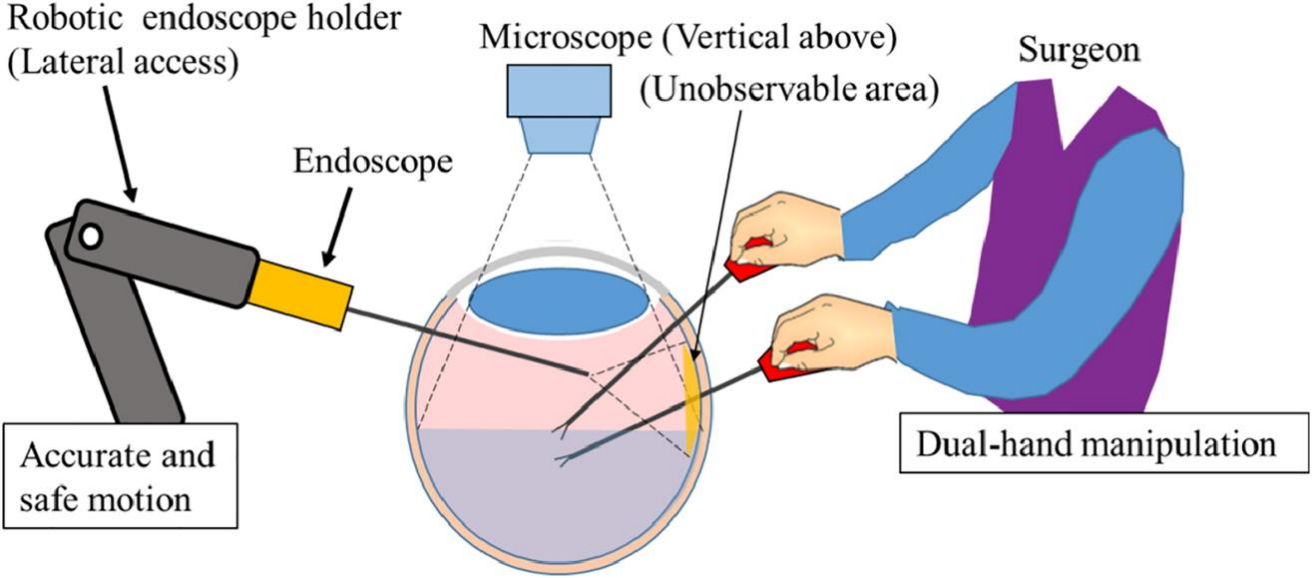
Application of a robotic endoscope holder for eye surgery

Tadano et al. developed a robotic laparoscope holder that allows the surgeon to intuitively adjust the laparoscope without the hand motion.^19^ Therefore, the authors apply the same concept of robotic holder to an endoscopic vitrectomy surgery and propose a novel robotic endoscope holder that will facilitate the endoscopic eye surgery in terms of liberating the surgeon's hand to allow dual‐hand operation. This device is expected to popularize endoscopic eye surgery. Figure 1 depicts the conceptual design of an endoscopic eye surgery performed using a robotic endoscope holder and a microscope. A slim‐sized robot arm must approach the surgical field near the surgeons' hand, stably holding an endoscope and precisely controlling the viewpoint according to the surgeons' command.

The structure of this paper is as follows. In Section 2, we discuss the requirements and the design of the proposed endoscope holder as well as some functional specifications. Section 3 presents the experimental results concerning each clinical requirement, and Section 4 presents the discussions and conclusions.

## 2 MATERIAL AND METHODS

### 2.1 Engineering and clinical requirements

The engineering requirements are related to the quality of design, which can be given as follows:

- **Easy sterilization**: As required by most of medical devices, the non‐sterilizable part (endoscope) must be easily separated from the sterilizable part (holder) for sterilization and tool exchange
- **Easy manual operability**: Similar to other surgical devices, the surgeon may have to manually move the end effector of the device; thus, it is expected that the device can be easily moved without large mechanical impedance

The clinical requirements are related to the compatibility of the design with respect to real surgical cases and can be given as follows:

- **Sufficient observable angle**: Because the surgeon uses an endoscope to view the places that cannot be viewed using a microscope, the robotic holder should allow the endoscope to provide views covering most of the inside area of the eyeball, regardless of the insertion direction. As a result, the required observable angles in the horizontal and the vertical perspectives are 90° and 80°, respectively.
- **Compatibility**: First, the installation of the robotic endoscope holder should not affect the layout of conventional operating rooms, wherein the surgical table (general width of 550–700 mm), the cannula equipment (size at the height of the surgical table is more than 500 mm), and the microscope (size at the height of the surgical table is more than 600 mm) are basically needed. The size of the additional endoscope holder should be lower than that of the existed devices as much as possible. Second, the end effector should be compact such that it does not interfere with the hand motion of the surgeon. Third, because the endoscope and microscopes are often used together, the device's workspace in the vertical direction should be 185–235 mm, which is the average focal distance of a standard surgical microscope.
- **Safety**: Two important aspects of medical safety were considered. First, the needle‐like endoscope must not fall and damage the retina when the surgeon accidentally released the endoscope during manual operation or the incautious power off situation occurred. Second, the motion of the robot holder should be stable and precise without applying large forces on the patient's eyeball. Based on the data obtained with respect to the behaviour of an expert surgeon, a sclera force of less than 120 mN is considered to be safe.^20^

### 2.2 Design of the robotic endoscope holder

**FIGURE 2:**
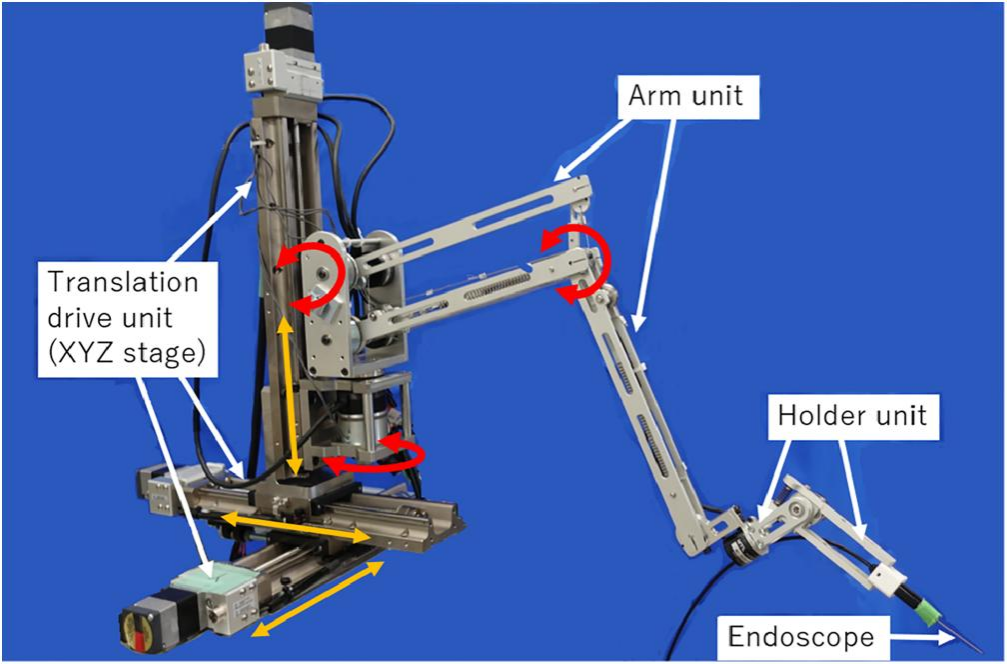
Appearance of the proposed robotic endoscope holder, that is, the Eye Explorer

Figure 2 presents an overview of the robotic endoscope holder, that is, the Eye Explorer, which has been developed to meet the aforementioned requirements. The Eye Explorer has eight degrees of freedom (DOFs) and comprises three main units: the translation drive, arm unit and holder units. The translation unit is active, whereas the arm unit is passive with mechanical brakes and the holder unit is passive. The detailed functions of each subunit are provided in the subsequent subsections.

#### 2.2.1 Translation drive unit

The translation drive unit is an XYZ stage with three DOFs (MODEL ARM24SAK; Oriental Motor Co. Ltd). Because this unit requires high stiffness and positioning accuracy to provide a stable view, the XYZ stage was selected. The maximum moving velocity in every axis is 80 mm/s, and the positioning accuracy and vibration magnitude at each axis are 0.01 and 0.005 mm, respectively. Moreover, the kinematics model for an XYZ system can be easily obtained.

#### 2.2.2 Arm unit

**FIGURE 3:**
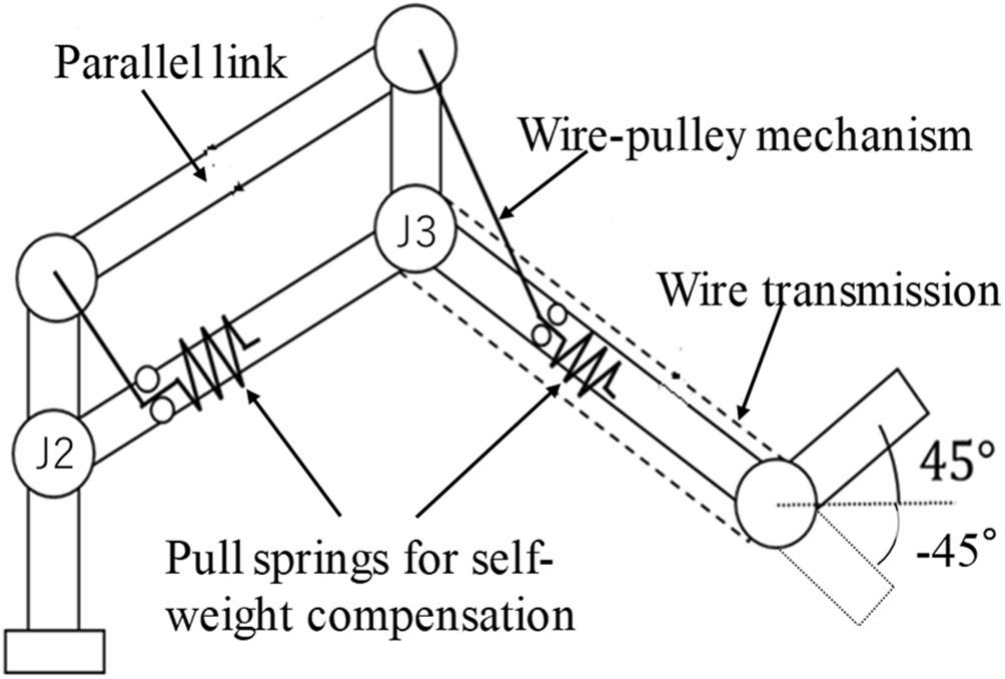
Schematic of the power transmission of the arm unit for self‐weight compensation

The arm unit has three DOFs of rotary joints. Joint J2 is a parallel link mechanism, whereas J3 is a serial wire‐linkage mechanism (the stainless wires were pre‐stretched before winding at the pulleys to eliminate the relative sliding between the wire and pulley). Figure 3 depicts a schematic of the linkage mechanisms of the arm unit. The angle of the base of the holder unit can be maintained constant because of the mechanical constraints with respect to the parallel link and the wire transmission mechanisms (the benefit of this design will be mentioned later). No actuator was installed in this unit, and electromagnetic brakes (112‐04, output torque: 1.2 N·m, MIKI PULLEY. Co., Ltd) were utilized to determine the orientation of each joint.

**FIGURE 4:**
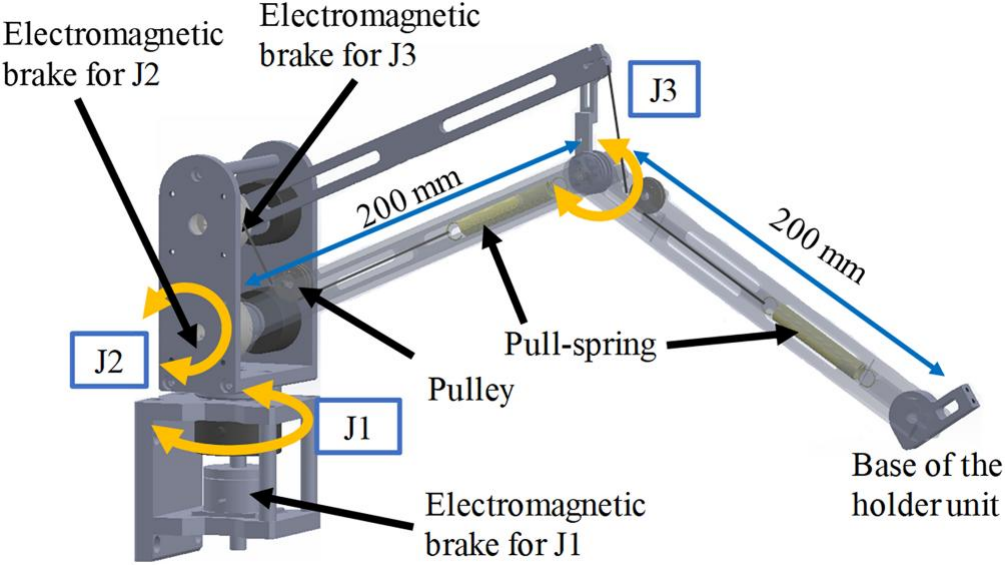
Design and overview of the arm unit

Additionally, we designed a self‐weight compensation mechanism using pull springs with wire‐pulley mechanisms (Figure 3). The necessary spring constants of the pull springs were determined based on the principle proposed by Yamada and Morita. ^21^ Then, we bought the pull‐spring with the determined spring constant and set them on the corresponding positions on the arm unit. Figure 4 also shows the pull‐springs inside the arm unit.

#### 2.2.3 Holder unit

**FIGURE 5:**
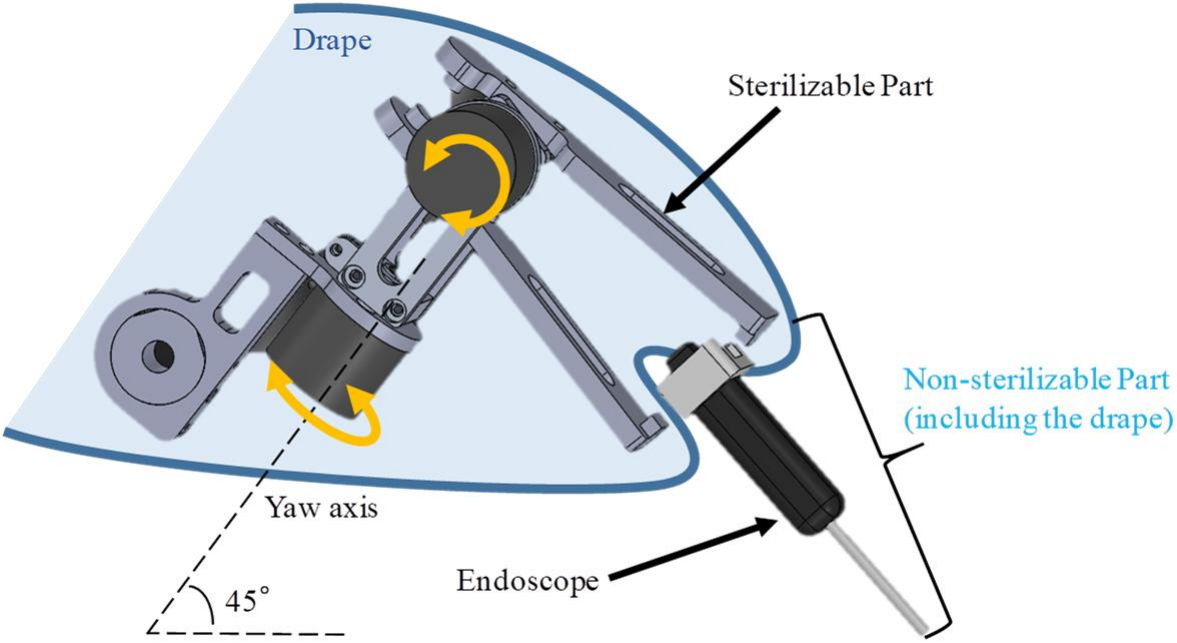
Holder unit mechanism and the manner in which it adapts to an endoscope

The holder unit is a passive gimbal mechanism with two DOFs, as shown in Figure 5. This passive mechanism was selected because of its compact and lightweight design without actuators. The benefits of the passive mechanism will be presented in Section 2.3. In addition, because of the linkage mechanisms of the arm unit, the angle of the yaw axis to the horizontal plane is maintained at 45° or −45°, which cannot only provide a sufficient operation space to the surgeon's hands but can also avoid a singular posture at which the endoscope, the base of the holder unit, and the second link are parallel. The posture in which moving the endoscope view left and right will exert a large force on the eye of the patient.

The holder unit uses a clip to attach the endoscope on a simple drape cover, as shown in Figure 5. This makes it easy to separate the non‐sterilizable part (endoscope) from the sterilizable part (holder unit), satisfying the engineering requirement of easy sterilization.

### 2.3 Operation method

**FIGURE 6:**
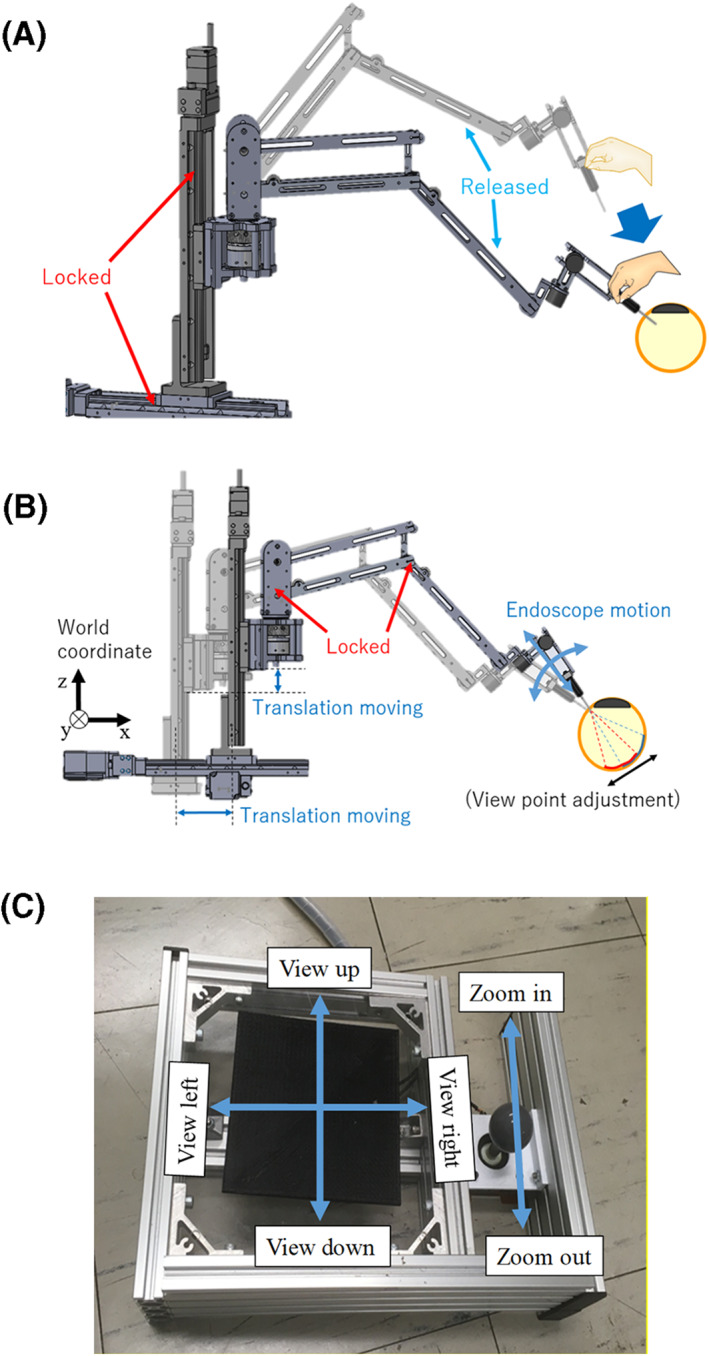
(A) Control scheme in the passive mode. (B) Control scheme in the active mode. (C) Footswitch for adjusting the endoscope view during operation

This device can be operated in two modes, that is, the passive mode and the active mode. Figure 6A, B depict the operation scheme of the Eye Explorer in these two modes, respectively. Table 1 presents the motion states of the three mechanical units in each operation mode.

**TABLE 1:** Motion states of each unit with the operation modes

	Passive mode	Active mode
Translation drive unit	Locked	Active
Arm unit	Free	Locked
Holder unit	Free	Free

In the passive mode, which is mainly considered before and after the surgery, the surgeon pulls the endoscope to the target position, inserts it into the eyeball through a trocar hole and adjusts the endoscope such that it points toward the area where the surgical operation will begin. In this mode, all motions are driven by the surgoen's hands. As shown in Figure 6A, the translation drive unit (XYZ stage) is locked in the passive mode, and the electromagnetic breaks in the arm unit are released, indicating that the arm unit can be freely moved by the surgeon. The arm unit was designed to allow easy manual positioning of the endoscope. As shown in Figure 3, the electromagnetic brakes and endoscopes for each joint were located at the base of the arm unit to reduce the weight of the movable section, and the released electromagnetic breaks have low mechanical impedance.

The surgeon will begin to operate on the patient's eye after inserting the endoscope into the eyeball and adjusting the view area. During the operation, the surgeon can use the active mode to alter the view provided by the endoscope. As shown in Figure 6B, in this mode, the electromagnetic breaks in the arm unit are locked and the whole arm unit is rigid. The posture and position of the passive endoscope holder unit are only determined by the translation motion of the XYZ stage.

Because the surgeon uses both hands to operate, we designed a footswitch (Figure 6C) that will allow the surgeon to intuitively adjust the endoscope view by foot. The black square at the left side is movable, and it is connected to a potentiometer that measures its motion direction. Thus, the endoscope camera, which shows the view on a screen, will move towards the direction where the surgeon moves the black square. The joystick at the right side determines the zooming of the endoscope view. Thus, the endoscope view can be adjusted without interrupting the hand operation.

Furthermore, if necessary, the surgeon can adjust the view by hand by switching to the passive mode. The two modes are alternated with another footswitch (not shown in Figure 6), with the device remaining in passive mode if the surgeon continuously steps on the footswitch. Hence, mode changing can be achieved easily by slightly uplifting one foot.

### 2.4 Control method

When the surgeon operates the foot switch in the active mode, the motion of the XYZ stage determines the velocity of the arm base (*V*_base_). The velocity of the gimbal centre at the holder unit relative to the coordinate system of the arm base (***V***_gim_) equals to ***V***_base_ because the arm unit with locked joints can be considered as a rigid body.

**FIGURE 7:**
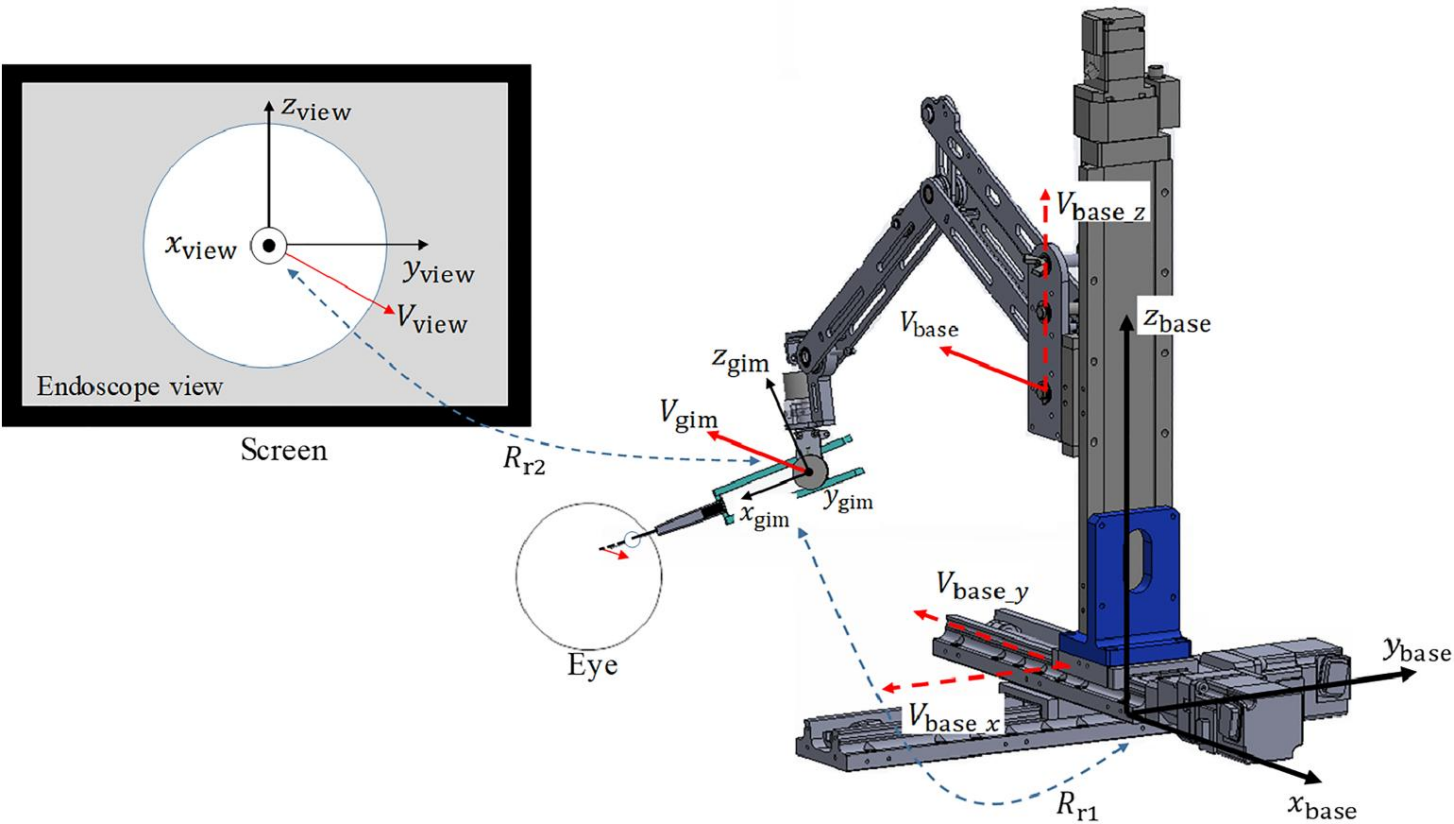
Relationship between motion velocity of the endoscope view and the XYZ stage

Figure 7 shows the coordinate systems of the screen view, endoscope, and arm base. The velocity of the gimbal centre relative to the coordinate system of the endoscope $\left(V_{\text{gim}}^{'}\right)$ determines the motion of the endoscope view shown on the screen (***V***_view_).

Therefore, a necessary ***V***_base_ required to generate the expected ***V***_view_ can be calculated by the following function:

(1) $$V_{\text{base}}=V_{\text{gim}}=R_{r1}\cdot V_{\text{gim}}^{'}=R_{r1}\cdot R_{r2}\cdot V_{\text{view}}\text{,}$$

where ***R***_r1_ is a transition matrix that changes the coordinate system of the endoscope to that of the base. Meanwhile, ***R***_r2_ is a transition matrix that changes the coordinate system of the screen into that of the endoscope. Furthermore, ***R***_r1_ is derived from the rotation angles at each joint, which are measured by the encoders (arm unit: MAH‐19‐524288N1; holder unit: MAH‐19‐524288N1, MTL lnc.).

We implemented a velocity control of the XYZ stage in each direction using ***V***_base_ in Equation (1) as the control reference. Moreover, the magnitude of ***V***_view_ is constant and was set in advance, while its direction is determined by the operator The control platform was developed is Ubuntu 18.04, and the period of the control loop was 0.001s. The accuracy of defining the position of the gimbal centre is 0.016 mm because the positioning accuracy of the XYZ stage at each axis was 0.01 mm. Considering a minimum duration time for an operator to input a control signal with his foot of approximately 0.05 s (50 loops), the resolution of the viewing angle in the real application was set to 0.7° to 1.3°, according to the insertion depth.

## 3 RESULTS

### 3.1 Manual operating force

In the passive mode, the arm and holder units should be easily movable so that the surgeon can concentrate on positioning the endoscope. Section 2 introduces some measures to reduce the mechanical impedance of the arm unit, including the implementation of self‐weight compensation using the released electromagnetic breaks and locating the brakes and the encoders at the base of the arm unit.

**FIGURE 8:**
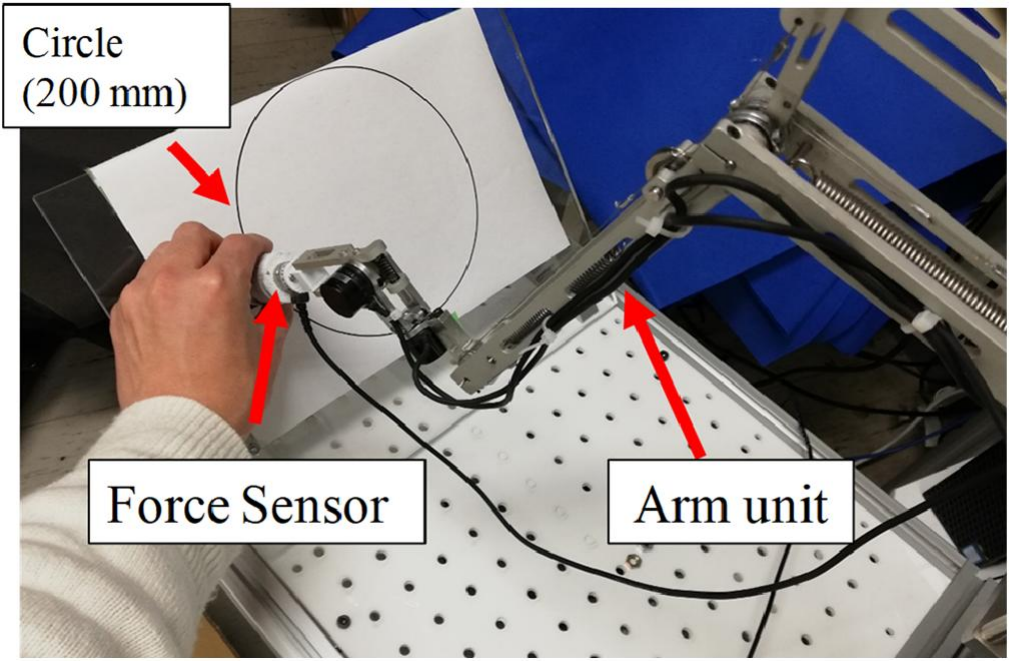
Experimental setup for measuring the operating force of the Eye Explorer

We implemented an experiment to confirm the force needed in the passive mode; a force sensor (Nano17, BI. AUTOTEC. LTD., resolution: 0.00625 N) was attached between the endoscope adaptor and the holder unit, and a reference circle with a diameter of 200 mm was prepared, as shown in Figure 8. Furthermore, one test operator was instructed to move the robot arm along the track of the reference circle while manually holding the endoscope within 5 s. The circle diameter of 200 mm is approximately the size of the upper hemisphere of a human head. The trajectory of this motion can cover most of the potential position where the operator moves the endoscope above the target eye. This motion was repeated four times. The test operator was required not to let the handle contact anything during the motion.

**FIGURE 9:**
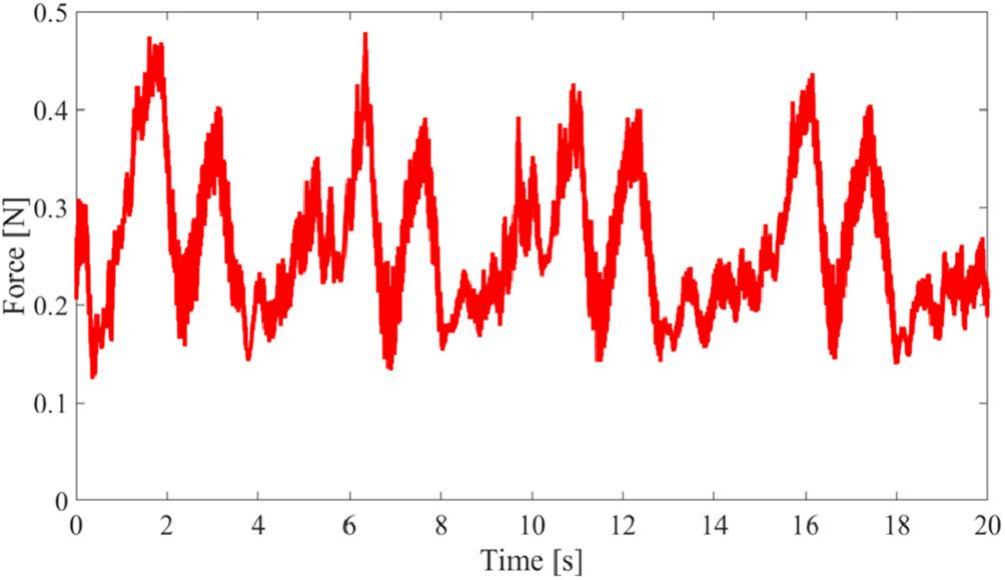
Magnitude of operating force during the manual circular movement

Figure 9 denotes the magnitude of the force measured by the force sensor. These results indicate that maximum and average values were 0.48 and 0.26 N, respectively. The required force for the operator to move the endoscope holder in the passive mode is similar to that used to hold a tool of 50 g, which would be considered lightweight by most people.

### 3.2 Working range and compatibility

**FIGURE 10:**
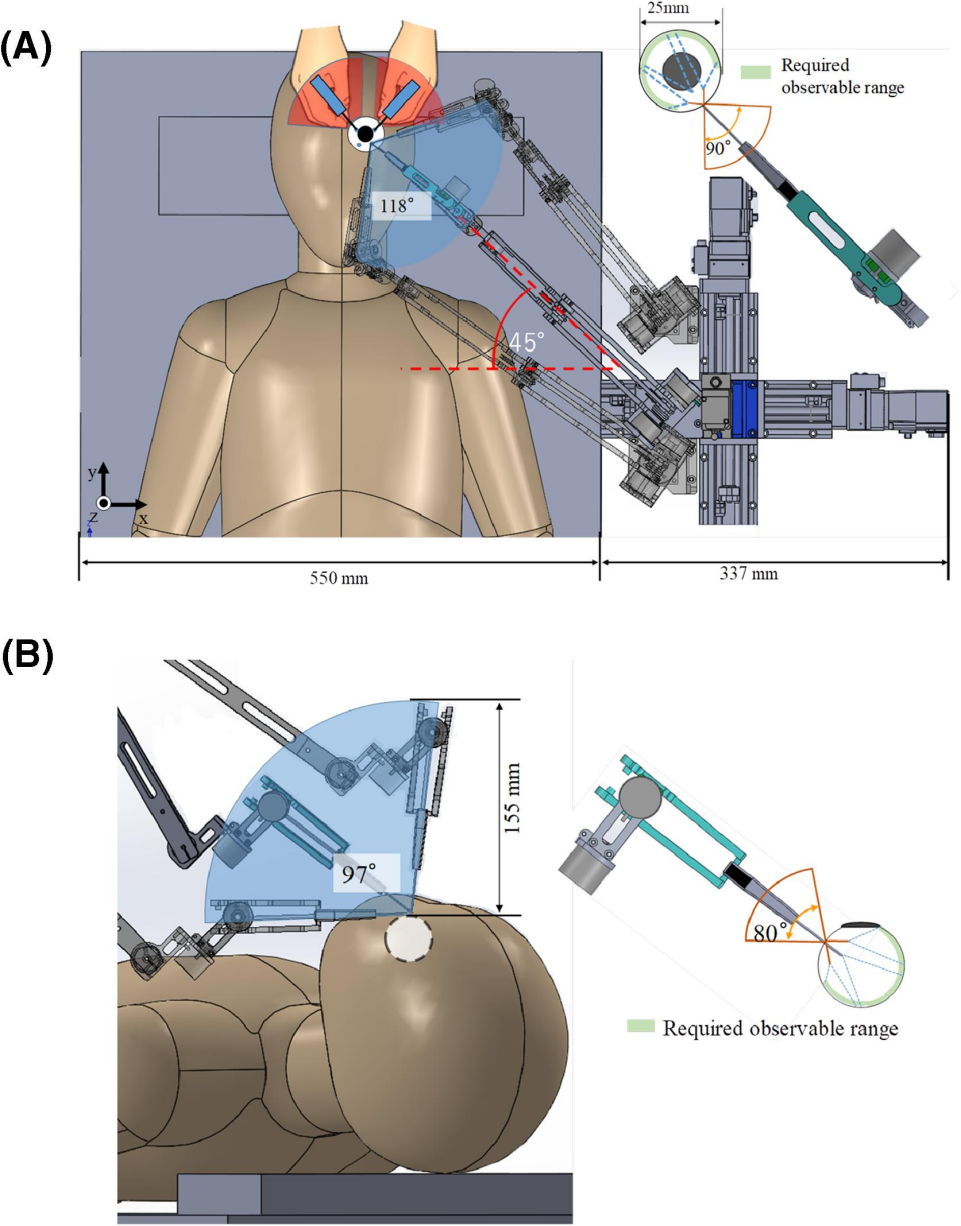
(A) Real and required observable ranges when using the endoscope holder during surgery, and the motion range of the hands of the surgeon (horizontal perspective). (B) Real and required observable ranges when using the endoscope holder during surgery (vertical perspective)

The Eye Explorer is mounted on a movable stand (wheels of the stand can be braked) next to the surgical table; the height of the movable stand can be adjusted according to that of the surgical table. The endoscope can access the objective eye from an angle of 45°. Figure 10 indicates the perspective range of the endoscope and the required perspective range for surgical cases, in which the endoscope holder and objective eye are on the same side of the patient's nose considering both the vertical and horizontal perspectives. The pitch angle of the endoscope in Figure 10A and the yaw angle in Figure 10B are maintained constant. The observable ranges of the endoscope in the vertical and horizontal perspectives are 118° and 97°, respectively.

**FIGURE 11:**
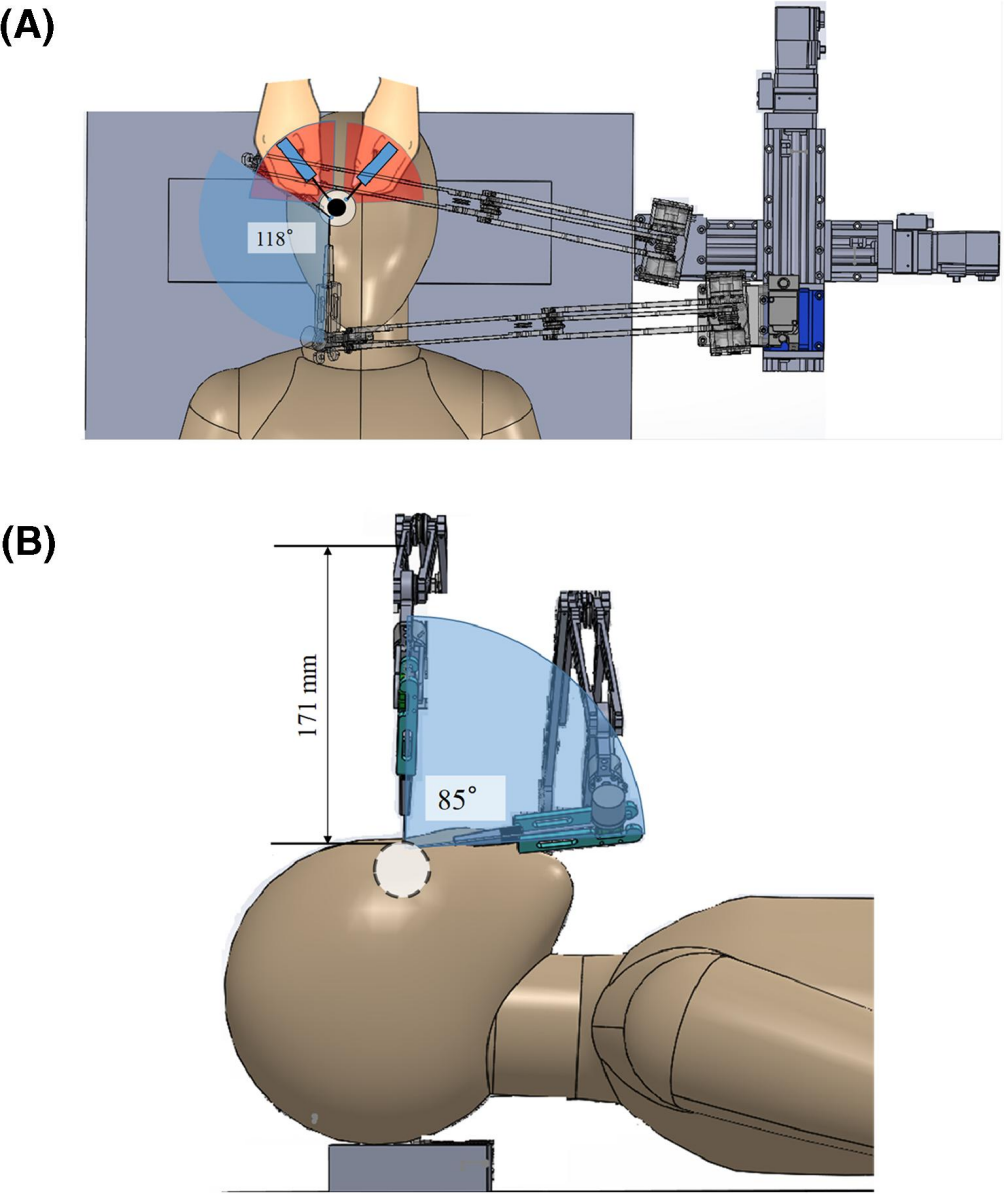
(A) Real and required observable ranges when using the endoscope holder during surgery, and the motion range of the hands of the surgeon (horizontal perspective). (B) Real and required observable ranges when using the endoscope holder during surgery (vertical perspective)

In cases that the endoscope holder and objective eye are on different sides of the patient's nose, the required and real observation range considering both perspectives are 118° and 85°, as shown in Figure 11 and the angle of the yaw axis of the holder unit relative to the horizontal plane is −45. Furthermore, the observable ranges considering both perspectives are larger than those required (90° and 80°), regardless of the access manner.

The red sectors in Figures 10 and 11 also show the necessary working range of the hands of the surgeon when facing the top of the forehead of the patient in most of the eye surgeries. Moreover, the maximum position above the patient's eye occupied by the endoscope holder is 155 and 171 mm.

### 3.3 Safety measurements

#### 3.3.1 Performance of self‐weight compensation

The needle‐like endoscope must not fall into the patient's eye. However, this may easily occur if the surgeon accidentally releases the device in the passive mode and could also occur in the active mode when the power to the electromagnetic brakes is cut off. Therefore, we developed self‐weight compensation for the device using the mechanism of the arm unit described in Section 2.2.2.

**FIGURE 12:**
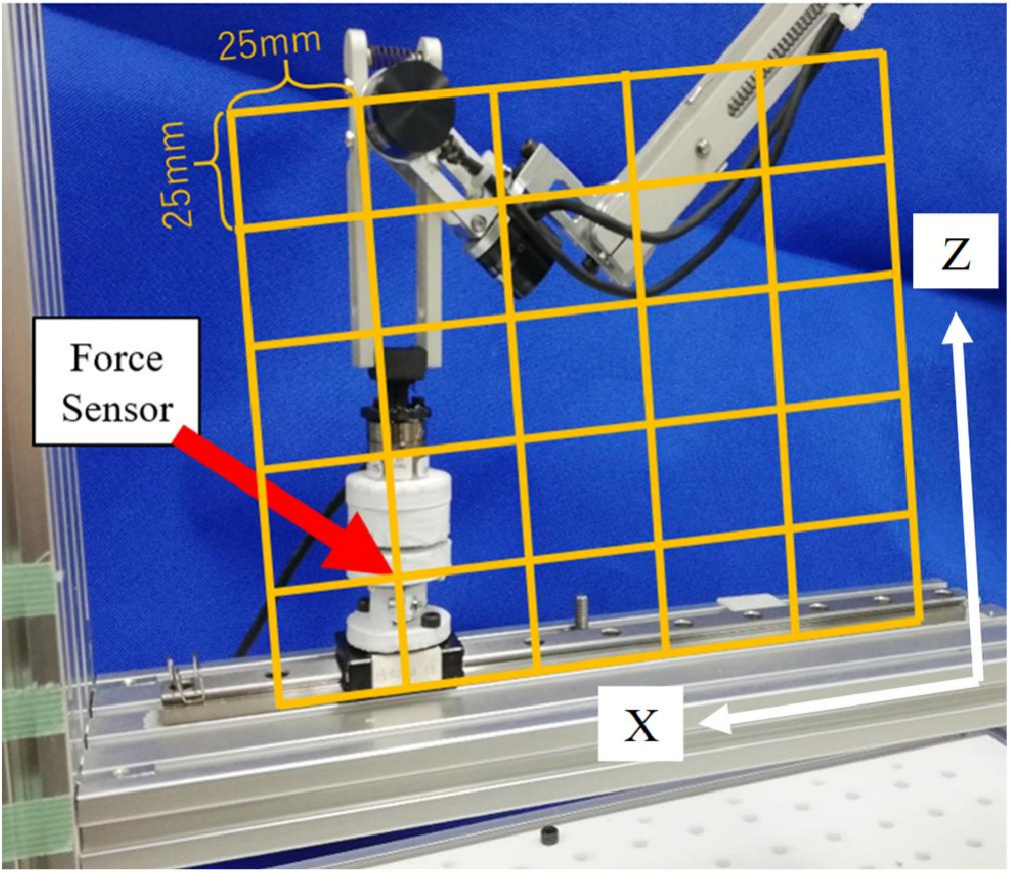
Experimental setup for evaluating the self‐weight compensation performance. The 36 points in the grid are the force measurement positions

This section introduces an experiment to evaluate the adopted self‐weight compensation. We measured the necessary force to keep the positon of the endoscope without external supporting. Figure 12 presents the experimental setup. The range of the yellow grid is the most common workspace (in the XZ plane) where the end effector appears when using the device. We selected 36 positions within the rectangle, which are the points where every horizontal and vertical line of the grid intersects. The distance between each adjacent evaluation points was 25 mm.

In the experiment, first, the tip of the holder unit was fixed at every evaluation point. Then, we released the electromagnetic brakes. The force senor (Nano17, BI. AUTOTEC. LTD., resolution: 0.00625 N), shown in Figure 12, measured the forces at each evaluation point. The measured force is also the force required to hold the positions at each evaluation point.

**FIGURE 13:**
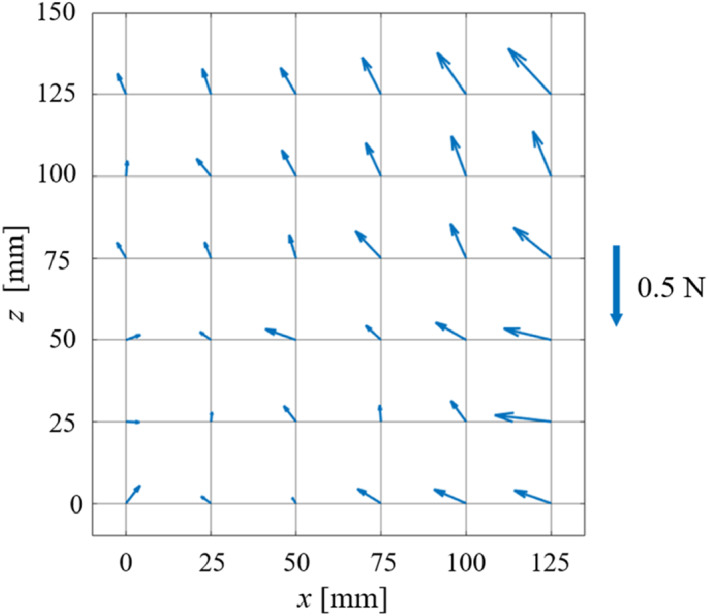
Vectors of the acting force measured at each point

Figure 13 presents the force vectors at each evaluation point obtained using the force sensor. The results indicated that the maximum necessary force to hold the designated positions was 0.46 N and that the mean force magnitude was 0.20 N. The directions of all the measured forces in the Z‐axis are upward. This experimental result demonstrates that the endoscope will not fall when the forces supporting the holder unit is accidentally released.

#### 3.3.2 External load exerted on the eye

When adjusting the endoscope view in the active mode, the interaction between the endoscope and insertion hole determines the external load exerted on the eye of the patient. This load should be small to ensure the security of the eye of the patient.

**FIGURE 14:**
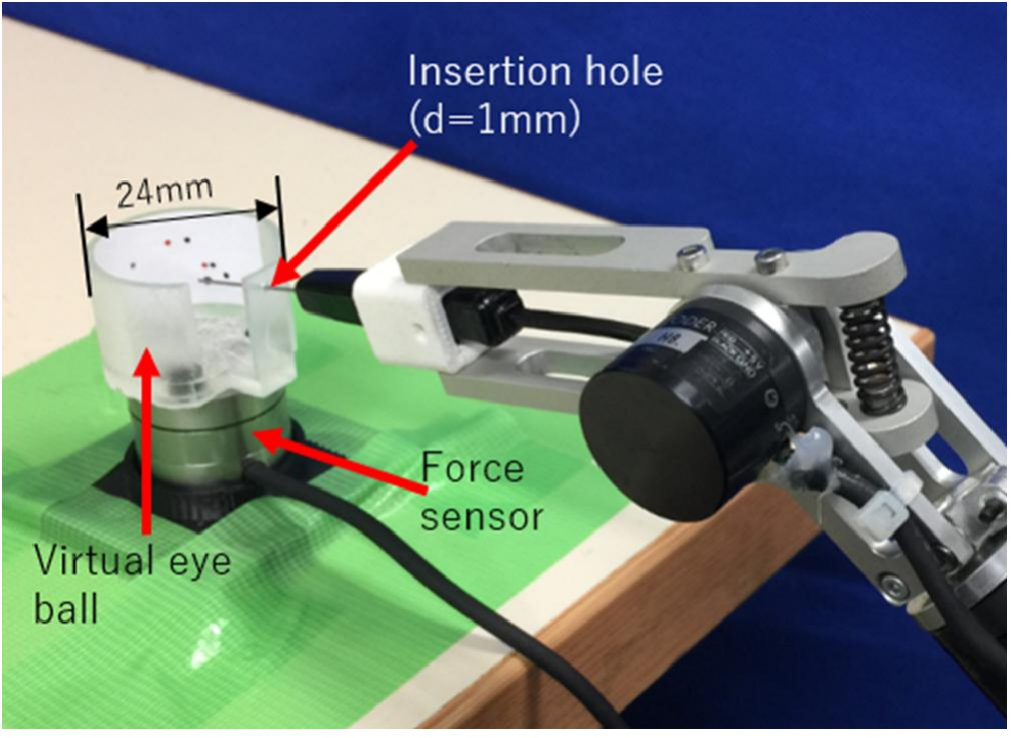
Apparatus used to measure the force exerted on the eye

**FIGURE 15:**
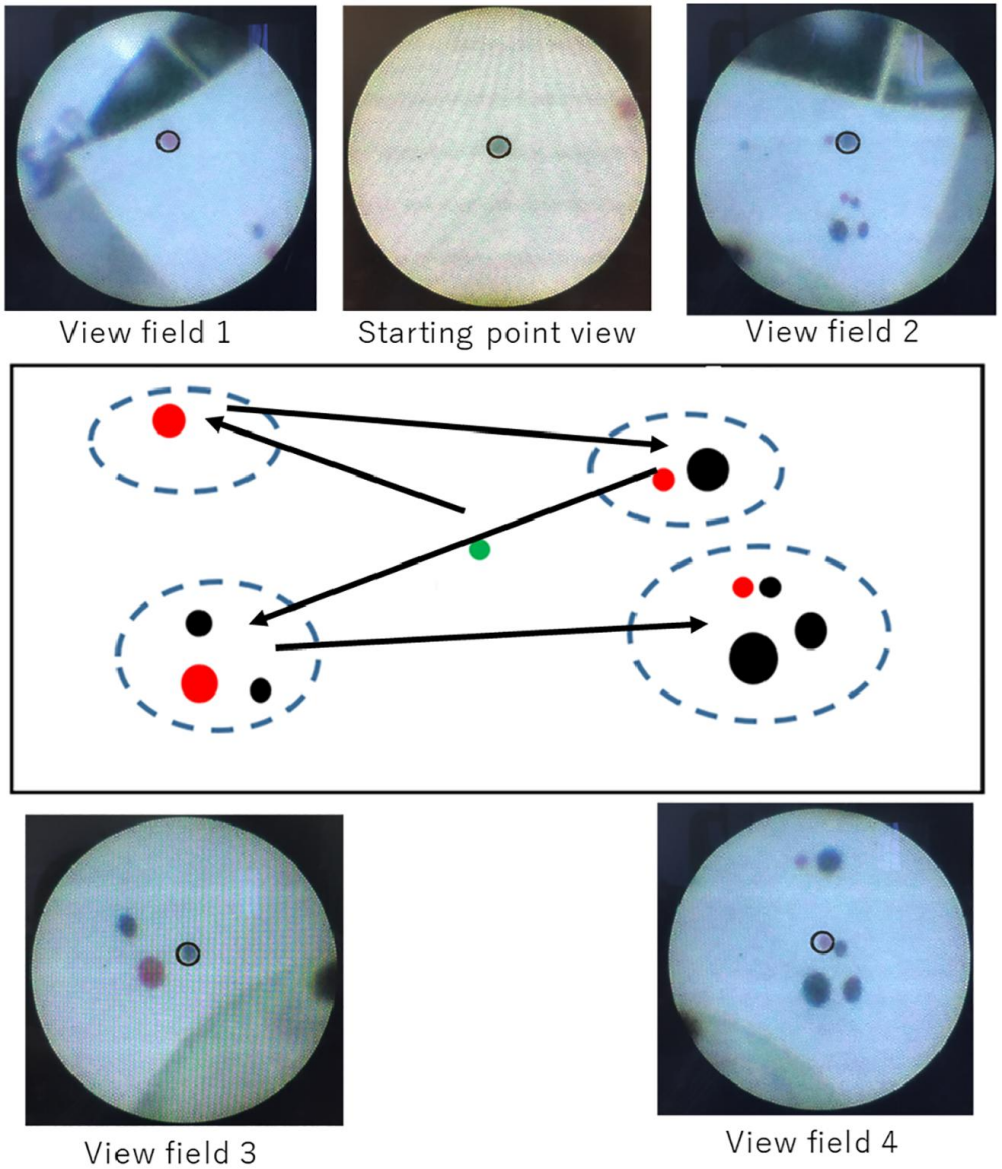
Layout of the four view fields inside the virtual eyeball and the corresponding endoscope view on the screen. The operators moved the viewpoint along the route shown by the arrows

To measure the force exerted on the patient's eye, a virtual eyeball with a diameter of 24 mm was made by 3D printing, on which an insertion hole with a diameter of 1.0 mm was set for the 23G needle‐like endoscope. Figure 14 depicts the designed experimental apparatus. A force sensor (MIRCO 4/20‐A, BL. AUTOTEC. LTD., resolution: 0.04 N) was mounted onto the virtual eyeball. Four view fields were assigned inside the virtual eyeball, as shown in Figure 15. A black ring was attached on the monitor screen as a sighting mark for view adjustment.

Five test operators moved the endoscope along the designated route, as shown in Figure 15, by operating the footswitch shown in Figure 6. All test operators were male engineer students of 21–25 years old, who did not operate the developed device before these experiments.

At each view field, the operators adjusted the viewpoint to ensure that the edge of each dot and the inner edge of the ring fit together. A series of view adjusting motions, including view translation and zooming, which are common during eye surgery, is required to complete this operation. The operators initially performed the viewpoint control task by holding the endoscope in their own hand. Then, they repeated the same task by operating Eye Explorer in the ‘active mode’. Each operator conducted the manual operation trial and the Eye Explorer (robotic) operation trial once after a brief practice of how to use the Eye Explorer.

**FIGURE 16:**
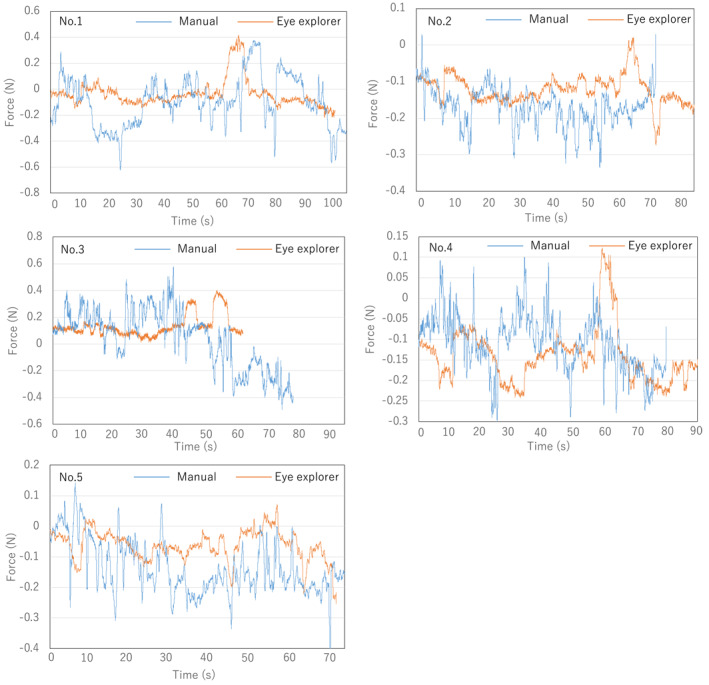
Force exerted on the virtual eye during the view adjustment task

Figure 16 presents the resulting time history of the measured force in the vertical direction when the five test operators were conducting the manual/robotic operation. Table 2 presents the comparison of the force data in case of all the subjects between the manual operation and the Eye Explorer operation with respect to the maximum value, the mean value and the standard deviation of the recorded force magnitude in the vertical direction.

**TABLE 2:** Comparison of the experimental data with respect to the exerted force in the vertical direction between manual and robotic operations

Test Operator	Max force (*N*)			Mean force (*N*)			Standard deviation (*N*)		
	Eye Explorer	Manual	Improvement rate (%)	Eye Explorer	Manual	Improvement rate (%)	Eye Explorer	Manual	Improvement rate (%)
No. 1	0.496	0.634	21.7	0.085	0.179	52.5	0.074	0.125	40.8
No. 2	0.285	0.381	25.1	0.127	0.169	24.9	0.042	0.057	26.3
No. 3	0.404	0.591	31.6	0.113	0.186	39.2	0.074	0.170	56.5
No. 4	0.241	0.298	19.1	0.130	0.149	14.6	0.051	0.064	20.3
No. 5	0.241	0.399	39.5	0.074	0.143	49.7	0.059	0.080	26.3

### 3.4 Evaluation of the operating time

Although a robotic endoscope holder may provide benefits, an increase in operation time would likely cause usability and implementation issues. Hence, we investigated the operating time of endoscope manipulation.

**FIGURE 17:**
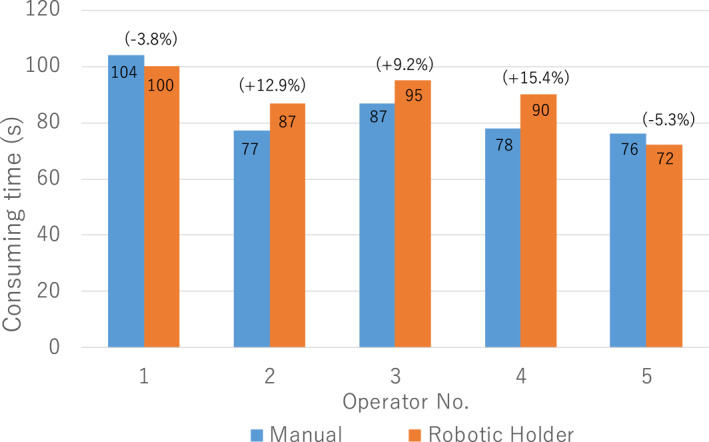
Comparison of the time spent to complete the view adjustment task in the manual and robotic operation methods

Figure 17 shows the comparison of the time spent to complete the view adjustment task between the manual and robotic operations described in Section 3.3.2. The pair corrections between the consumption time of two operation manners are high because every pair of data was generated from the same test operator. Hence, the paired student's *t*‐test was feasible despite the small sample size.^22^ The null hypothesis of the paired student's *t*‐test was: the average consumption time of the manual operation ${\bar{t}}_{\text{man}}$ equals that of the robotic operation ${\bar{t}}_{\text{rob}}$ $\left(H_{0}:{\bar{t}}_{\text{man}}-{\bar{t}}_{\text{rob}}=0\right)$. The significant level was 5%. The option of standard deviation was set unknown.

The testing result showed that |t| = 1.41, which is out of the rejection region of |t| ≥ 2.57. Hence, we can accept the null hypothesis, which means there are no difference in consumption time between the manual and robotic operations. Moreover, the moving speed of the Eye Explorer was set to 15 mm/s at the endoscope tip; the operators were instructed to move at normal speed during manual operation.

## 4 DISCUSSION

As introduced in Section 3.1, the required force applied by the operator to move the endoscope holder in the passive mode is similar to that used to hold a tool of 50 g, which would be considered lightweight by most people. Therefore, the engineering requirement of easy manual operability can be satisfied.

By calculating the visible area through the endoscope when using this device, the endoscope view covered 70% of the intraocular area, regardless of the insertion manner. Thus, the clinical requirement of a sufficient observable range is satisfied.

Considering the compatibility of the device, first, the width of the XYZ stage (337 mm) was less than two‐third of the surgical table (550 mm). Second, the necessary working range of the hands of the surgeon (red sectors) in Figures 10A and 11A may overlap with those of the endoscope holder (blue sectors). However, when the endoscope moves to the place overlapping the red sectors, the space between the arm unit and the eye of the patient remains more than 150 mm in the vertical direction, which is sufficient for the hand motion of the surgeon. As a result, the probability in which the hands of the surgeon conflicts with the endoscope holder unit was low. Third, the maximum workspace measurements of the holder unit above the patient's eye were 155 and 171 mm, which are within the requirements of the device workspace considering the vertical direction (185–235 mm) when using a surgical microscope; therefore, the motion of this device will not interfere with the operation. Moreover, the space above the head of the patient, which is occupied by the device, was smaller than that occupied by the device developed by Nassari,^2^ the IRISS,^3^ the KU Leuven robotic system 6 and the Preceyes surgical system,^7^ all of which are representative surgical robot systems for eye surgery. Therefore, the clinical requirement of compatibility is satisfied.

An ideal self‐weight compensation requires that the position of the endoscope is preserved in situations of power off. In this case, the forces measured by the force sensor should be zero. However, it is difficult to realize the ideal self‐weight compensation because of the weight uncertainties (cable, tiny parts, etc.). Hence, we overestimated the weight in the design of the equipment. The real effect of the self‐weight compensation in the upward forces is shown in Figure 13. The upward forces may retract the endoscope out from the patient eye; the force exerted on the eye during the retraction be focused in future works because the upward force may let the endoscope retrieve from the patient's eye.

The comparison between the force exerted on the eye in manual and robotic view adjustment is shown in Figure 16. The Eye Explorer reduced the maximum and mean forces exerted on the eye and fluctuation of force on the insertion hole. Furthermore, Table 2 shows that all indexes were improved by at least 15% for all test operators by using the Eye Explorer. Moreover, the endoscope view shown on the screen showed no noticeable vibration because of the vibration magnitude of the XYZ stage (0.005 mm at each axis), which is lower than that of a surgeon's hand (approximately 0.1 mm^2^). Therefore, the clinical requirement of safety is satisfied.

The force shown in Figure 16 is the sclera force during the operation, which is an important safety quantity in eye surgery. Table 2 shows that the average sclera forces are lower or near the safe threshold of 0.12 N.^21^ However, the maximum forces are larger than 0.12 N. Note that the elasticity of the 3D printed eye used in this experiment is different from that of a real eye. The elasticity of tissue may reduce the maximum forces, which will be confirmed in future works. Moreover, the sclera force is related to the motion velocity of this device, which can be reduced whenever necessary.

Regarding the operation efficiency, the comparison result of the operation time only considered the process of the endoscope manipulation, not the total operation time of eye surgery. Now that this device does not impair the efficiency of the endoscope manipulation, and the overall operational efficiency is believed to be improved by considering the improved dexterity of the dual‐hand operation realized by the Eye Explorer.

## 5 CONCLUSION AND FUTURE WORKS

In this study, we developed a novel robotic endoscope holder for conducting eye surgeries named Eye Explorer that can hold an endoscope instead of the surgeon's hand. Using the Eye Explorer, the endoscope view can be adjusted without using the surgeon's hand. Hence, this device is expected to enable a dual‐hand operation during endoscopic eye surgeries and popularize the endoscopic eye surgery.

The experimental results demonstrated that the Eye Explorer satisfies the engineering requirements. Here, the mechanism makes it easy to place a sterilized cover on the robotic arm and separate the non‐sterilizable part (endoscope) from the sterilizable part (holder unit). The lightweight mechanical design allows the operator to manipulate this device in the passive mode using forces that are lower than 0.5 N.

The device satisfied the established clinical requirements. The size of the base was less than two‐third of the conventional instruments, such as the surgical table. Furthermore, the observable range of the endoscope is larger than that required in both the vertical and horizontal perspectives, ensuring that the endoscope view covered 70% of the intraocular area. The motion of the endoscope holder is not likely to interfere with the motion of the surgeon's hands and microscope. The safety measures were also considered, with the self‐weight compensation mechanism, preventing the needle‐like endoscope from falling and avoiding unintended damage to the retina. Moreover, the external load exerted on the eyeball when adjusting the endoscope view was considerably reduced.

Furthermore, the efficiency of the endoscope view adjustment by using this device did not differ from that of manual manipulation.

In future work, we intend to make several improvements to this device for clinical applications. First, an automatic instrument tracking system will be incorporated into the Eye Explorer to realize a fully automated assistive function and improve the direct user control interface. Second, we will consider more safety measures, including emergency measures when unexpected movement between the patient's eye and the endoscope happens (the patient's head moves or a large knocking is exerted on the arm unit), endoscope insertion depth control,^23^ workspace registration, and a motion restriction function. Furthermore, we will evaluate the leaning process of using this device by analysing the feedback from real surgeons.

## CONFLICT OF INTEREST

The authors do not have any conflict of interests to declare in relation to this manuscript.
